# Continuous scavenging of broadband vibrations via omnipotent tandem triboelectric nanogenerators with cascade impact structure

**DOI:** 10.1038/s41598-019-44683-5

**Published:** 2019-06-03

**Authors:** Divij Bhatia, Hee Jae Hwang, Nghia Dinh Huynh, Sangmin Lee, Choongyeop Lee, Youngsuk Nam, Jin-Gyun Kim, Dukhyun Choi

**Affiliations:** 1 Department of Mechanical Engineering, Kyung Hee University, 1732 Deogyeong-daero, Giheung-gu, Yongin-si, Gyeonggi-do 17104 South Korea; 2 School of Mechanical Engineering, Chung-Ang University, 84 Heukseuk-ro, Dongjak-gu, Seoul, 06974 South Korea

**Keywords:** Devices for energy harvesting, Mechanical engineering

## Abstract

Ambient vibration energy is highly irregular in force and frequency. Triboelectric nanogenerators (TENG) can convert ambient mechanical energy into useable electricity. In order to effectively convert irregular ambient vibrations into electricity, the TENG should be capable of reliably continuous operation despite variability in input forces and frequencies. In this study, we propose a tandem triboelectric nanogenerator with cascade impact structure (CIT-TENG) for continuously scavenging input vibrations with broadband frequencies. Based on resonance theory, four TENGs were explicitly designed to operate in tandem and cover a targeted frequency range of 0–40 Hz. However, due to the cascade impact structure of CIT-TENG, each TENG could produce output even under non-resonant conditions. We systematically studied the cascade impact dynamics of the CIT-TENG using finite element simulations and experiments to show how it enables continuous scavenging from 0–40 Hz even under low input accelerations of 0.2 G–0.5 G m/s^2^. Finally, we demonstrated that the CIT-TENG could not only scavenge broadband vibrations from a single source such as a car dashboard, but it could also scavenge very low frequency vibrations from water waves and very high frequency vibrations from air compressor machines. Thus, we showed that the CIT-TENG can be used in multiple applications without any need for redesign validating its use as an omnipotent vibration energy scavenger.

## Introduction

Triboelectrification is a ubiquitous and naturally occurring phenomena that occurs when two different materials come into contact and rub against each other. The effect can be constructively used for various applications such as nano-patterning^1^, self-assembly^2^, recycling^3^, smoke filtration^4^, and mechanical energy harvesting using triboelectric nanogenerators (TENGs)^5^. Due to the increasing demand for renewable energy, there has been considerable interest shown in TENG based energy harvesting systems by the research community, and several approaches ranging from material processing to structural design have been proposed to improve and regulate its output^6–8^.

Vibration energy harvesters based on TENGs rely on resonance design, that is, the natural frequency of the TENG vibration system should approximately equal the input source vibration frequency. The natural frequency of the TENG in turn depends on parameters such as mass and stiffness which can be selected based on target frequency^8^. Furthermore, under non-linear impact the output frequency response of a vibration TENG stiffens near the resonance region resulting in a broadband output which depends on level of input forces and gap distance between the moving and fixed layers of the TENG^9,10^. It is important for a vibration energy harvester to have broadband operation since ambient vibration energy is highly irregular in force and frequency. In order to increase the operation bandwidth, the idea of tandem TENGs was proposed by the authors where helical coil spring based vibration TENGs were explicitly designed to operate at specific target frequencies, and then stacked together as one system to achieve the desired bandwidth coverage^10^. Similar tandem systems have been proposed for cantilever beam spring based vibration TENGs^11^ and fixed-fixed beam spring based vibration TENGs^12^. However, under low input vibration forces the typical tandem TENGs have gaps in their output frequency response resulting in non-continuous scavenging. Previously some research works have proposed frequency up-conversion structures for piezoelectric^13^ and electromagnetic^14^ vibration energy harvesters. In these frequency up-conversion structures, two vibration systems are employed, one designed to oscillate at low frequency and the other at high frequency. When the low frequency vibration system is in resonance, it impacts with the high frequency vibration system resulting in the translation of low frequency oscillations into high frequency oscillations. Similarly, high frequency oscillations can be translated into low frequency in frequency down-conversion structures. In this work we show that by adopting ideas from frequency up-conversion structures we can achieve continuous scavenging even under low input vibration forces. Furthermore, not only do different ambient vibration sources have different frequencies, but even a single vibration source can have ever changing attributes. For example, sea waves typically have very low frequencies, but they can change their features as they approach the shore^15^; car vibrations, such as on the front dashboard or the rear deck can depend on suspension design, road conditions and the car speed^16^; other machine vibrations such as from engines or motors can have several frequency components at low as well as high frequencies^17^. Thus, in this work we propose an all-purpose and omnipotent vibration energy harvester that can operate continuously under any variations in the properties of the vibration input source.

We investigate a tandem TENG with cascade impact structure (CIT-TENG) that shows continuous operation within target frequency range even under low input vibration forces. The CIT-TENG uses four layers of TENGs such that the resonating TENG impacts with the next TENGs thereby producing a domino impact effect. Due to cascade impact, the TENGs that are non-resonating can also produce output thereby contributing the bandwidth broadening. The non-linear dynamics of the CIT-TENG were analyzed using finite element simulation as well as experimentally. The continuous scavenging of CIT-TENG under different levels of input forces was verified and compared to the non-continuous scavenging of a typical tandem TENG. Finally, omnipotence of the CIT-TENG was demonstrated by using the same device for harvesting low frequency vibrations from water waves, high frequency vibrations form an air compressor machine, and broadband frequency vibrations from a car dashboard.

## Results and Discussion

**Figure 1:**
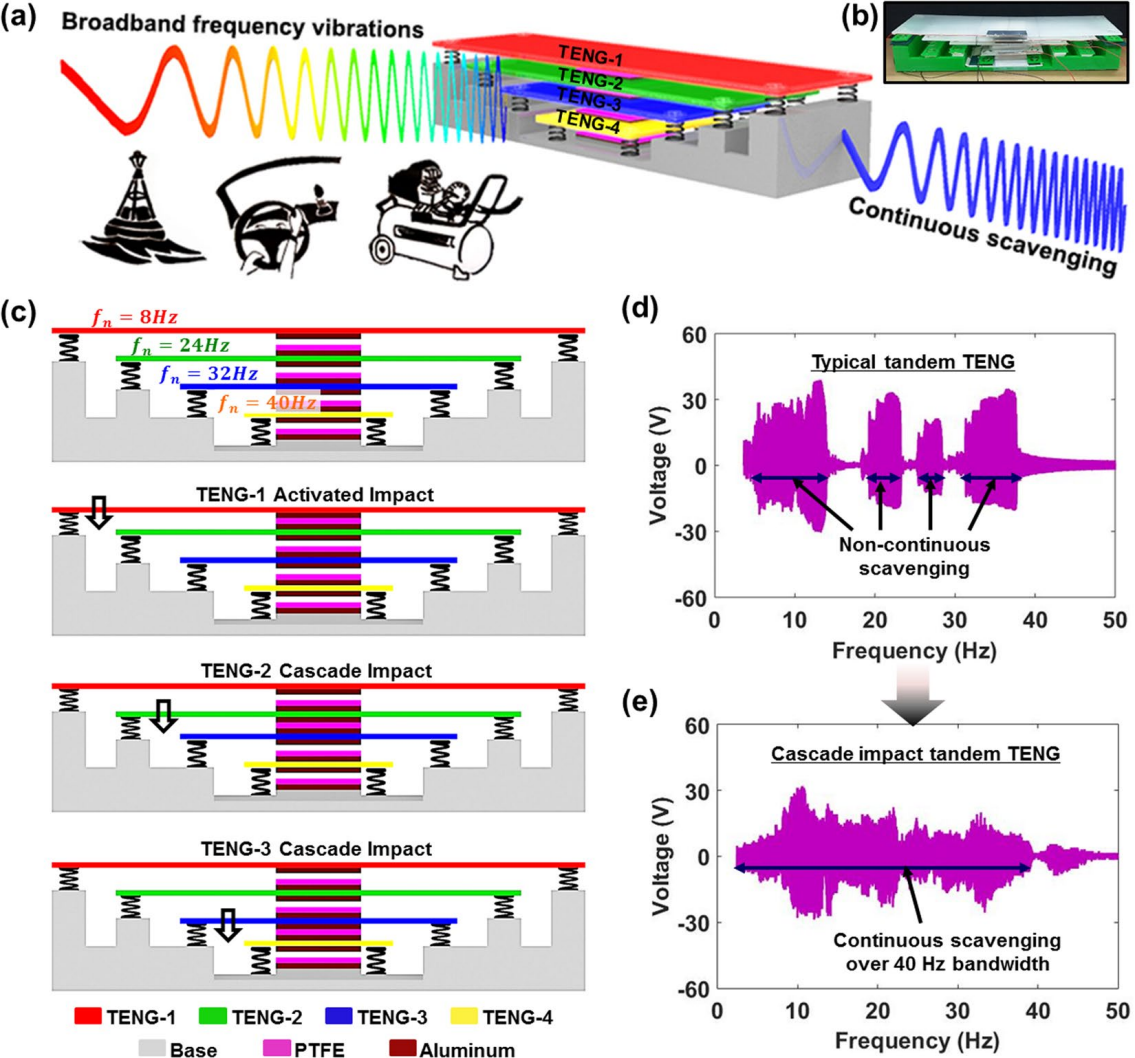
Basic idea behind CIT-TENG. (**a**) Conceptual illustration showing that CIT-TENG can continuously scavenge broadband frequency vibrations from multiple ambient sources. (**b**) Photograph of actual CIT-TENG developed in this work. (**c**) Cross-section schematic of CIT-TENG design and the cascade impact behavior initiated by activated TENG-1. (**d**) Voltage output frequency response of typical tandem TENG showing non-continuous scavenging at input acceleration of 0.2 G m/s^2^. (**d**) Voltage output frequency response of CIT-TENG showing continuous scavenging within 40 Hz bandwidth at input acceleration of 0.2 G m/s^2^.

Figure 1a conceptually illustrates that the CIT-TENG can continuously scavenge broadband frequency vibrations from multiple ambient sources such as water waves, car dashboard, and air compressor machines. The reader is pointed to Supporting Fig. S1 for construction and operation of a basic vertical contact separation mode TENG. The CIT-TENG employs four such vertical contact separation mode TENGs as shown in the photograph Fig. 1b and cross-section schematic in Fig. 1c. The TENGs were each explicitly designed to resonate at natural frequencies of 8 Hz, 24 Hz, 32 Hz, and 40 Hz, respectively. Figure 1c also illustrates the cascade impact mechanism, where the resonating motion of activated TENG-1 causes impact with TENG-2 which propagates through the rest of the structure resulting in impact between TENG-2 and TENG-3, and between TENG-3 and TENG-4. Figure 1d shows the limitation of a typical tandem TENG structure for scavenging irregular frequency vibrations. The voltage output frequency response shows non-continuous scavenging under low input acceleration of 0.2 G m/s^2^. Supporting Note 1 describes the fabrication and assembly of the simply stacked structure type typical tandem TENG, Supporting Fig. S2 shows its photograph and Supporting Fig. S3 shows its frequency response under input accelerations of 0.2G-0.5 G m/s^2^. It was observed that under the higher input accelerations of 0.4 G m/s^2^ and beyond, the output frequency response could be adequately continuous, however under low input accelerations of 0.2 G–0.3 G m/s^2^, there were significant gaps in the response where the output was zero. This is because in this simply stacked structure of the typical tandem TENG, each TENG vibrates in solitary fashion with no interaction with the other TENGs. The CIT-TENG, on the contrary, has considerable interactions between the individual TENGs, and thus shows continuous energy scavenging within the targeted frequency range of 40 Hz even under low input acceleration of 0.2 m/s^2^ as shown in Fig. 1e. Detailed photograph of the CIT-TENG is shown in Supporting Fig. S4 and its frequency response under input accelerations of 0.2G–0.5 G m/s^2^ is shown in Supporting Fig. S5. As the input acceleration level of the vibration shaker was increased from 0.2 G m/s^2^ to 0.5 G m/s^2^, the CIT-TENG output levels also increased, and no gaps were observed in the response. Thus, the proposed CIT-TENG structure is superior to the previously proposed tandem TENG structure since it can operate continuously within the targeted frequency range even under low input accelerations.

The design of each vibration TENG involved the consideration of its natural resonance frequency as well as the bandwidth broadening due to impact stiffening. If the vibration TENG mass and stiffness are represented as *m* and *k*, respectively, then based on the resonance equation, the TENG natural frequency is given as, $2\pi {f}_{n,sys}=\sqrt{k/m}$. Accordingly, in order to achieve the target natural resonance frequencies of 8 Hz, 24 Hz, 32 Hz, and 40 Hz for each of the TENGs, their mass and stiffness values were determined as 40 grams and 25*4 N/m for TENG-1, 30 grams and 160*4 N/m for TENG-2, 38 grams and 400*4 N/m for TENG-3, and 26 grams and 400*4 N/m for TENG-4, respectively. The reader is pointed to our previous work^10^ for details on how to select the mass and stiffness values. In this study, we employed finite element simulation using ABAQUS/CAE software to determine the vibration dynamics of each TENG. However, for completeness, the vibration equations of motion for the individual TENGs during no-impact and impact conditions is described in Supporting Note 2. Supporting Movie S1 shows the simulated vibration dynamics of the CIT-TENG structure at sinusoidal input frequencies of 10 Hz, 20 Hz, 30 Hz, and 40 Hz, under an input acceleration on 0.5 G m/s^2^. The first point of observation from the movie is that different TENGs are actively resonating during each of these input frequencies, related to the natural resonance frequency of the respective TENG. The second point of observation is that the motion of a single TENG initiates cascade impact behavior amongst adjacent TENGs making them vibrate at their non-resonant frequencies. The simulated displacement time response of each TENG at 10 Hz, 20 Hz, 30 Hz, and 40 Hz input frequencies is provided in Supporting Fig. S6. It can be observed from the simulation results that not only do the resonating TENG (corresponding to the input frequency) show high amplitude motion, but also the non-resonating TENGs show significantly large amplitudes due to cascade impact initiation.

**Figure 2:**
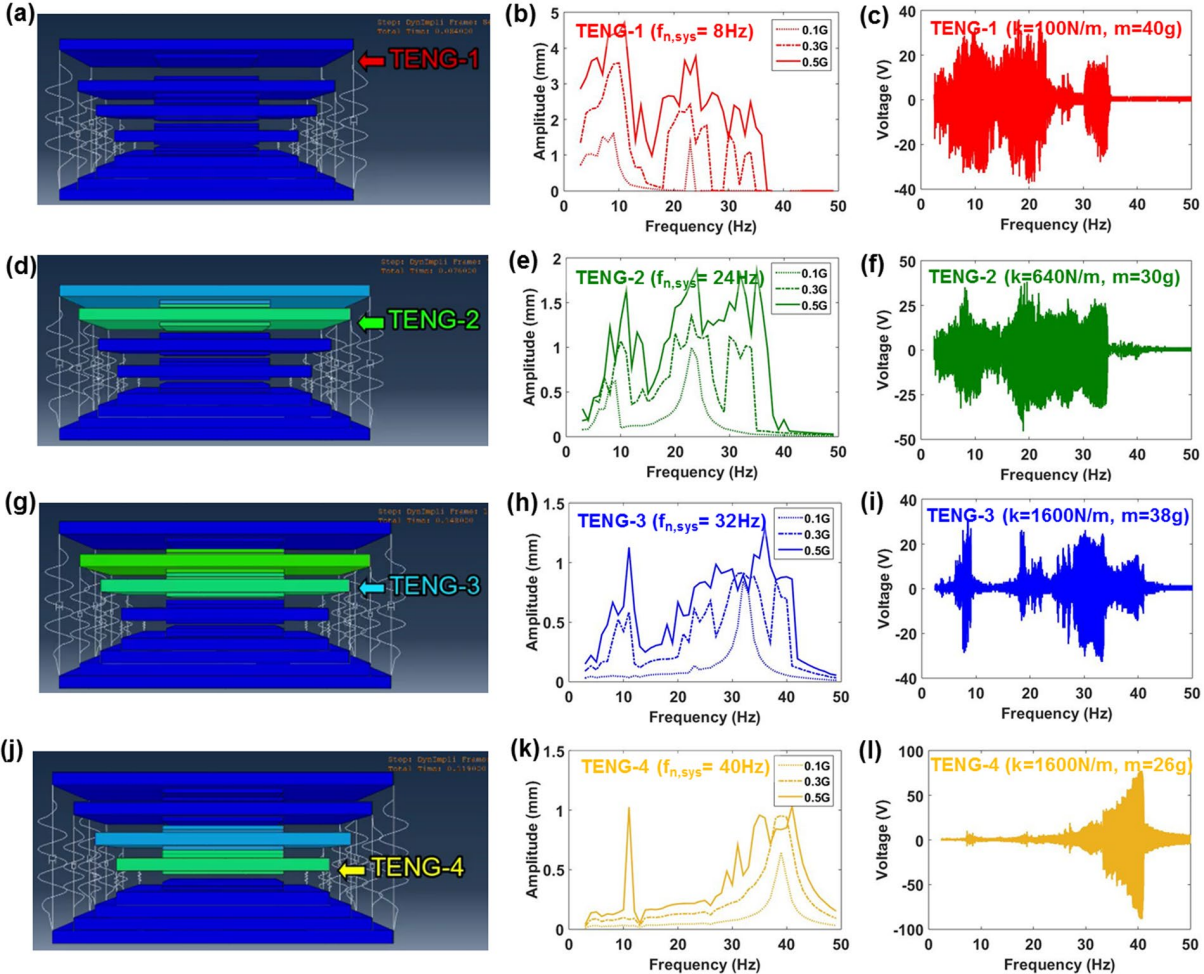
TENG dynamics in the cascade impact structure. Simulation image indicating resonance, simulated amplitude frequency response results, and voltage output frequency response results of TENG-1 (**a–c**) respectively, TENG-2 (**d–f**) respectively, TENG-3 (**g–i**) respectively, and TENG-4 (**j–l**) respectively. The simulated amplitude frequency response results are shown for input accelerations of 0.1 G m/s^2^, 0.3 G m/s^2^, 0.5 G m/s^2^. The voltage output frequency response results are shown for input acceleration of 0.5 G m/s^2^.

In order to better understand the dynamic behavior each TENG at their resonating and non-resonating frequencies, their frequency responses were simulated and validated experimentally. Figure 2a shows simulation image from Supporting Movie S1 indicating the actively resonating vibration of TENG-1. Figure 2b shows the simulated amplitude frequency response results for TENG-1 under input accelerations of 0.1 G m/s^2^, 0.3 G m/s^2^, and 0.5 G m/s^2^. Figure 2c shows the corresponding voltage output frequency response results for TENG-1 under input acceleration of 0.5 G m/s^2^. It can be seen from the simulation and experimental results that even though the natural resonance frequency of TENG-1 is 8 Hz, due to cascade impact the TENG-1 output can be high even at non-resonant frequencies such as when TENG-2 and TENG-3 are in resonance, due to frequency down-conversion behavior. Furthermore, the simulated amplitude response and experimental voltage response results are reasonably well matched, validating the finite element simulation method. Figure 2d shows simulation image indicating the actively resonating vibration of TENG-2, which can be expected to directly impact both TENG-1 and TENG-3. Thus even though the natural resonance frequency of TENG-2 is 24 Hz, the TENG-2 also shows high amplitude and voltage at the non-resonant frequencies when TENG-1 and TENG-3 are in resonance, due to frequency up-conversion and frequency down-conversion, respectively, as shown in Fig. 2e,f. Next, the simulation image Fig. 2g indicates the actively resonating vibration of TENG-3, which can be expected to directly impact TENG-2 and TENG-4. Here TENG-3 not only shows high amplitude and voltage output at its own resonant frequency (32 Hz) and the resonating frequencies of TENG-2 (due to frequency up-conversion) and TENG-4 (due to frequency down-conversion), but also the resonating vibration of TENG-1 contributes to the output of TENG-3 due to cascade impact and frequency up-conversion, as shown in Fig. 2h,i. Finally, the simulation image Fig. 2j indicates the actively resonating vibration of TENG-4, which is expected to directly impact only TENG-3. Thus apart from high amplitude and voltage response at its own natural frequency (40 Hz), its output is also influenced by TENG-3 vibration due to frequency up-conversion, as shown in Fig. 2k,l. Based on these results we conclude that the cascade impact structure induced each TENG to show significant vibration not only at their respective natural resonance frequencies, but even at non-resonant frequencies which become more prominent as the input acceleration levels were increased. This behavior ultimately helps obtain continuous scavenging within targeted frequency range from CIT-TENG.

**Figure 3:**
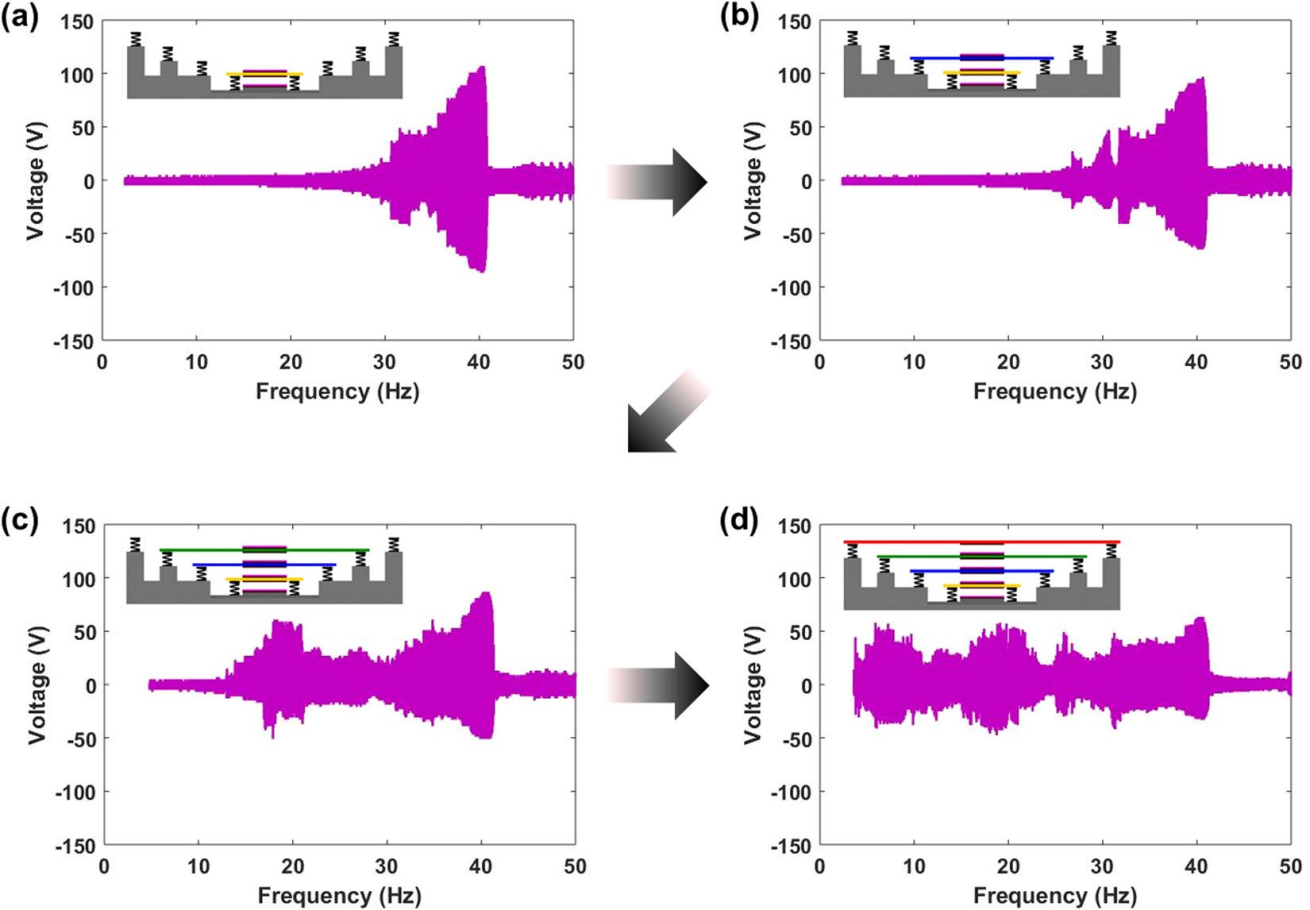
Layer by layer installing of TENGs. Voltage output frequency response results from CIT-TENG with (**a**) only TENG-4, (**b**) TENG-3 added above TENG-4, (**c**) TENG-2 added above TENG-3, and (**d**) TENG-1 added above TENG-2.

Further analysis of the CIT-TENG system was performed by studying the effect of layer by layer installing of TENGs. Figure 3 shows that as each additional TENG is installed above the previous TENG, the output gradually broadens at the expense of the output level. Figure 3a shows that only TENG-4 will produce resonating output at 40 Hz. When TENG-3 is added above TENG-4 as in Fig. 3b, they not only vibrate at their resonating frequencies but also interact with one another. The motion of TENG-4 is restricted by TENG-3 which results in the reduction of its output level. However it now also experiences double impact which has been shown to broaden the TENG bandwidth^18^. When TENG-2 is added above TENG-3 as in Fig. 3c, cascade impact initiation begins to take its effect. Now the TENGs not only operate at their resonating frequencies but also at the resonating frequencies of the other TENGs as discussed previously. This further contributes to smoothly broadening out the output frequency response. Finally when TENG-1 is added above TENG-2 as in Fig. 3d, continuous operation from 3–40 Hz could be observed. Supporting Fig. S7 tracks the decrease in TENG-4 output voltage at 40 Hz input frequency, as TENGs are installed layer by layer above it. The rate of output voltage decrease from TENG-4 was determined as 14 V per TENG installed. Supporting Movie S2 shows the actual vibration dynamics of the individual TENGs during CIT-TENG frequency response. As the input frequency was increased from 3 Hz to 50 Hz, the resonating TENG can be observed to show the maximum amplitude motion which in turn effects the motion of adjacent TENGs through cascade impact. Apart from up-down motion, other vibration mode motion such as the sideways rocking motion can be observed for TENG-1 and TENG-2 at frequencies higher than their resonant frequencies. These additional vibration modes have lower amplitude vibrations so they can only significantly contribute to the output when the gap distance between the TENGs is reduced. Thus for the CIT-TENG used in application demonstration, the gap distances between the TENGs were slightly reduced to make use of these additional vibration modes which positively contributed to the continuous scavenging behavior of CIT-TENG, as previously shown in the voltage output frequency response results in Supporting Fig. S5.

**Figure 4:**
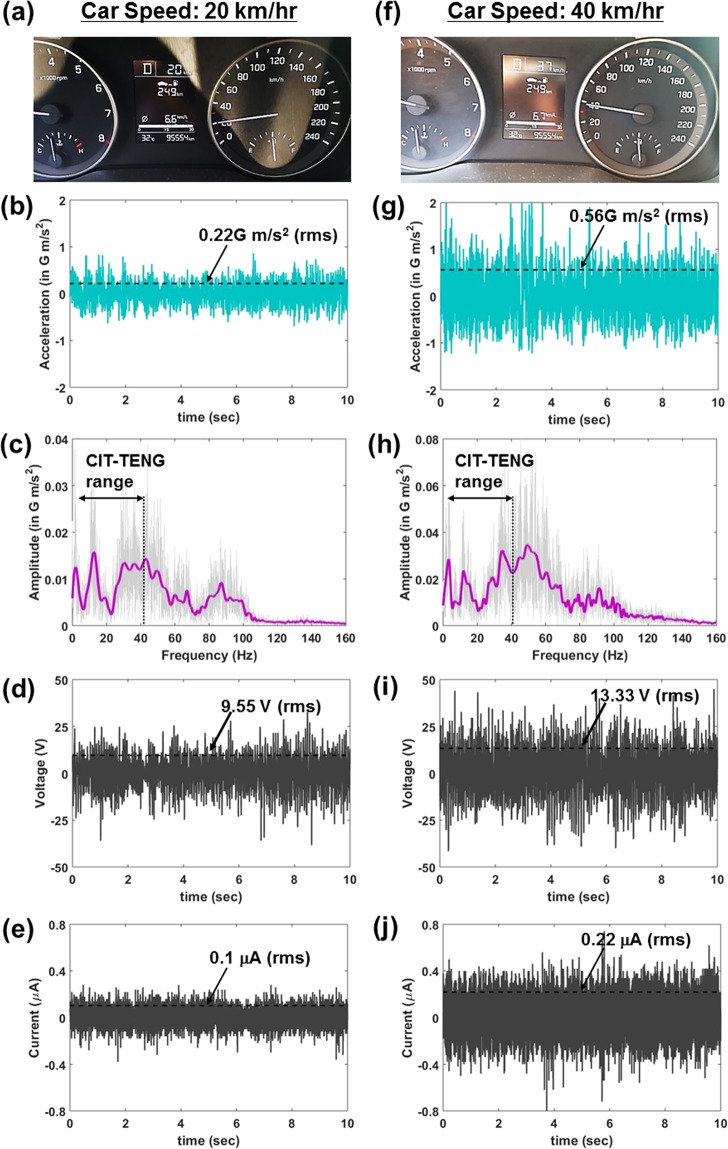
CIT-TENG as broadband frequency vibration energy scavenger. Car driving speed of 20 km/hr: (**a**) speedometer photograph, (**b**) input acceleration measurement, (**c**) input frequency spectrum with raw data (grey) and smoothing curve (magenta), (**d**) CIT-TENG output voltage results, and (**e**) CIT-TENG output current results. Car driving speed of 40 km/hr: (**f**) speedometer photograph, (**g**) input acceleration measurement, (**h**) input frequency spectrum with raw data (grey) and smoothing curve (magenta), (**i**) CIT-TENG output voltage results, and (**j**) CIT-TENG output current results.

In order to demonstrate application of CIT-TENG as broadband frequency vibration energy scavenger, it was placed on a car dashboard and experimental measurements were taken at driving speeds of 20 km/hr and 40 km/hr. Figure 4a shows photograph of the car speedometer at 20 km/hr driving speed. At this speed, the input vibration acceleration was measured to be around 0.22 G m/s^2^ (rms) as shown in Fig. 4b. The input frequency spectrum was obtained by taking fast fourier transform (FFT) of the input acceleration data, following which smoothing function was used to make the input vibration frequencies easier to identify. Figure 4c shows that the input frequency spectrum of the car dashboard vibrations was broadband with resonance peaks observed at 2 Hz, 12 Hz and 30–50 Hz. The voltage and current of the CIT-TENG under these input conditions were 9.55 V (rms) and 0.1 μA (rms) as shown in Fig. 4d,e, respectively. FFT of the voltage output in Supporting Fig. S8a showed that the CIT-TENG scavenged the input vibrations of 12 Hz and 30–40 Hz frequencies. Figure 4f shows photograph of the car speedometer at 40 km/hr driving speed. At this speed, the input vibration acceleration was measured to be around 0.56 G m/s^2^ (rms) as shown in Fig. 4g. The corresponding input frequency spectrum had resonance peaks at the same frequencies as 20 km/hr driving speed. However, compared to the frequency spectrum at 20 km/hr, the higher frequencies of 30–50 Hz were more dominant at 40 km/hr as shown in Fig. 4h. The voltage and current of the CIT-TENG at 40 km/hr were 13.33 V (rms) and 0.22 μA (rms) as shown in Fig. 4i,j, respectively. The increase in the CIT-TENG output levels at 40 km/hr compared to the output at 20 km/hr was attributed to the increase in the input acceleration level. FFT of the voltage output at 40 km/hr in Supporting Fig. S8b showed that the CIT-TENG could scavenge the more dominant higher frequency components observed in the input frequency spectrum. Thus we showed that input vibrations from even a single vibration source can be broadband in nature, and that in order to effectively scavenge broadband frequency vibrations from such ambient sources we require continuous scavenging capability as shown by the CIT-TENG.

**Figure 5:**
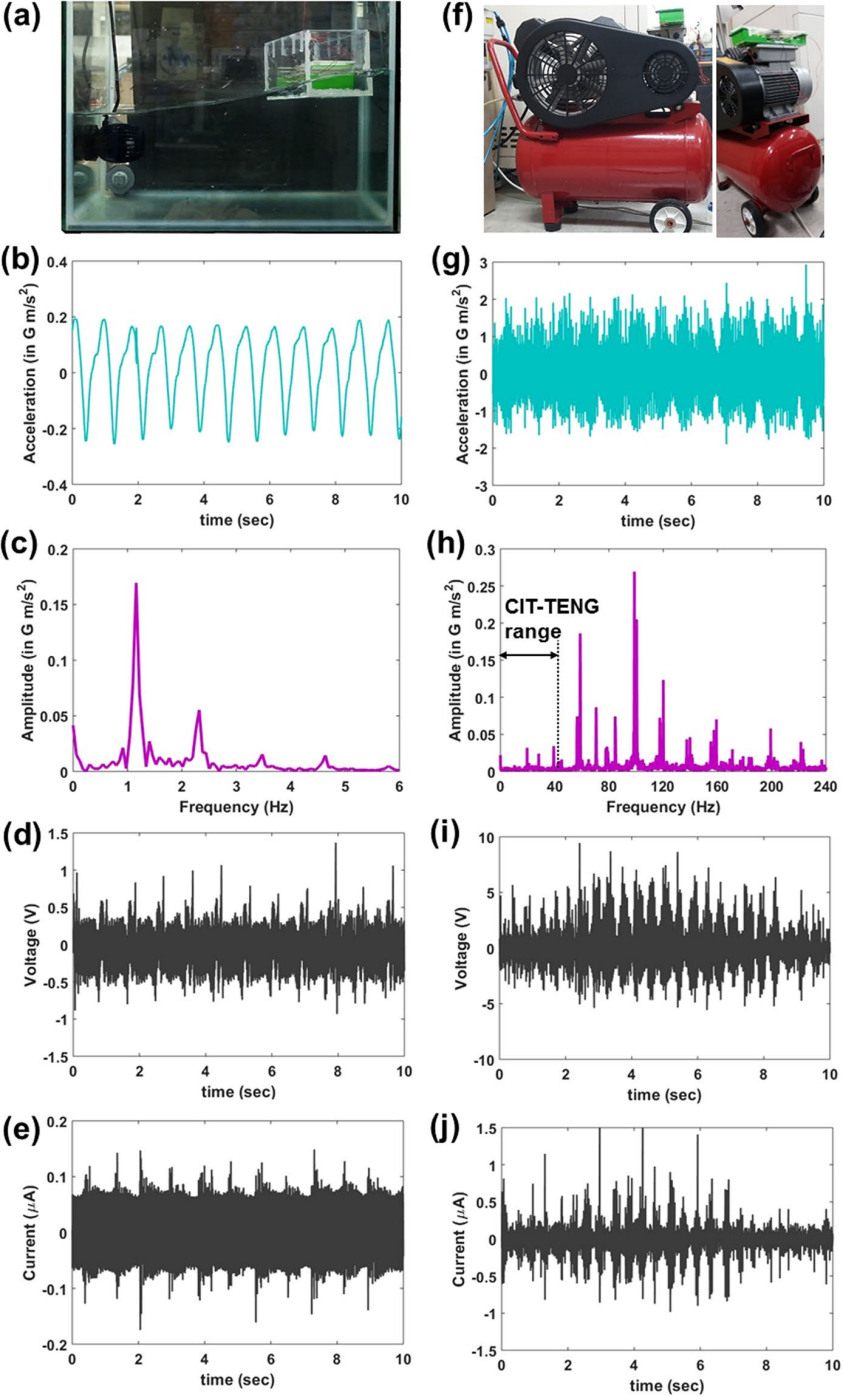
CIT-TENG as omnipotent vibration energy scavenger. Water wave vibration energy scavenging: (**a**) photograph of experimental setup, (**b**) input acceleration measurement, (**c**) input frequency spectrum, (**d**) CIT-TENG output voltage results, and (**e**) CIT-TENG output current results. Air compressor vibration energy scavenging: (**f**) photograph of experimental setup, (**g**) input acceleration measurement, (**h**) input frequency spectrum with CIT-TENG operation range indicated, (**i**) CIT-TENG output voltage results, and (**j**) CIT-TENG output current results.

In order to demonstrate CIT-TENG as an omnipotent or multi-purpose vibration energy scavenger, it was used for scavenging low frequency water wave vibrations and high frequency air compressor machine vibrations. Figure 5a shows photograph of the experimental setup for water waves vibration energy scavenging. The input vibration acceleration from the waves was measured to be around 0.2 G m/s^2^ as shown in Fig. 5b. The corresponding input frequency spectrum had resonance peak at about 1 Hz as shown in Fig. 5c. The voltage and current output of the CIT-TENG from the water waves was found to be about 0.75 V and 0.1 μA as shown in Fig. 5d,e, respectively. FFT of the voltage output in Supporting Fig. S9a showed prominent peak at 1 Hz, similar to the input frequency spectrum. Figure 5f shows photograph of the experimental setup for air compressor vibration energy scavenging, where the CIT-TENG was affixed on top of the motor housing. The input vibration acceleration was measured to be around 1.5 G m/s^2^ as shown in Fig. 5g. The corresponding input frequency spectrum had multiple resonance peaks with the highest peak at 100 Hz as shown in Fig. 5h. However, since the CIT-TENG operating bandwidth was up to 40 Hz as indicated in the figure, only the resonance peaks at 20 Hz, 30 Hz and 40 Hz could be expected to activate the TENGs. The voltage and current output of the CIT-TENG from the air compressor was found to be about 5 V and 0.75 μA as shown in Fig. 5i,j, respectively. FFT of the voltage output in Supporting Fig. S9b showed prominent peak at 40 Hz, similar to the input frequency spectrum. The reader is pointed to Supporting Movie S3 which shows the CIT-TENG dynamics during car dashboard, water wave and air compressor application experiments. Through these three applications we demonstrated the effectivity of CIT-TENG as an omnipotent vibration energy scavenger.

The usage of CIT-TENG in powering practical applications requires an analysis of the output voltage, output current, and output power under electrical loading conditions. Supporting Fig. S10 provides these results at 10 Hz, 20 Hz, 30 Hz, and 40 Hz input frequencies for an input acceleration of 0.5 G m/s^2^. The output voltage (*V*) and output current were experimentally measured at different loading resistances (*R*), whereas output power (*P*) was determined using the formula, *P* = *V*^2^/*R*. The maximum power point was found to be at 5 MΩ impedance for 10 Hz input frequency, and at 10 MΩ impedance for 20 Hz, 30 Hz, and 40 Hz input frequencies. Supporting Movie S4 shows feasibility of using the CIT-TENG in powering practical applications. Indicator LEDs could be directly powered by the CIT-TENG when driven by an air compressor. Since the LEDs would only light-up when the air compressor was running, they could be used to indicate that the machine is in ON state.

In summary, we studied a tandem triboelectric nanogenerator with cascade impact structural design that enabled continuous scavenging of ambient vibrations with broadband frequencies within a targeted frequency range even at low input accelerations. We demonstrated that our CIT-TENG could scavenge broadband vibrations from a single vibration source in a car dashboard under variable driving speeds. Finally, we demonstrated that the CIT-TENG can be reliably used for different vibration energy scavenging applications such as scavenging low frequency water wave vibrations and high frequency air compressor machine vibrations without any need for redesign. The CIT-TENG is thus an omnipotent solution for all vibration energy scavenging applications.

## Methods

### CIT-TENG fabrication and assembly

The CIT-TENG was made up of four explicitly designed TENGs with target natural frequencies of 8 Hz, 24 Hz, 32 Hz, and 40 Hz. For each TENG, a polytetrafluoroethylene (PTFE) film of thickness 130 μm was used as the insulating dielectric material, and an aluminum film of thickness 16 μm was used as the top and bottom electrode material. The top electrode aluminum also served as the counter tribological contact material for static charging the PTFE surface. Effective contact area of the tribologically active top aluminum electrode and PTFE was 3.5 cm × 3.5 cm. This metal-insulator combination is commonly used as the active materials for TENGs based on the triboelectric series^6,19,20^. The substrates of each TENG as well as the base structure of the CIT-TENG were 3D printed out of polylactic acid (PLA). Commercial double-sided tape (polyethylene (PE) foam) was used to fix the top and bottom aluminum electrodes to the PLA substrates. Four springs were used on each corner of the PLA substrates to support the TENG mass and ensure appropriate gap distance was maintained between the tribologically active materials in the initial state. The length, width and height of the CIT-TENG were 25 cm, 7 cm and 5.5 cm respectively as shown in Supporting Fig. S4.

### Finite element simulation

FEA simulations of the CIT-TENG structural dynamics were performed using ABAQUS/CAE software by Dassault Systemes. A gap distance of 1 mm was maintained between the TENGs for all simulations. Isight software from Dassault Systemes was used to automate the simulation for determining the amplitude frequency response of each TENG under different input accelerations. In order to obtain the amplitude frequency response, the maximum amplitude of each TENG at input frequencies of 3 Hz to 50 Hz, in steps of 1 Hz, was determined and plotted.

### Frequency response testing and output measurement

The CIT-TENG output voltage was measured using Tektronix MDO3022 mixed domain oscilloscope with an input impedance of 40 MΩ. The current output was measured using Stanford Research Systems SR570 low noise current preamplifier connected to the Tektronix oscilloscope. The CIT-TENG frequency response tests were performed using Labworks Inc. ET-139 permanent magnet shaker that was powered using Labworks PA-138 linear power amplifier and controlled using m + p VibPilot hardware and VibControl software. The input signal to the shaker for the frequency sweep was selected as sinusoidal; the start/stop frequencies of 3 Hz (spec limit for the testing equipment) to 50 Hz as well as the input acceleration levels of 0.2 G m/s^2^ to 0.5 G m/s^2^ were selected using the VibControl software.

### Car dashboard, water wave, and air compressor vibration testing

The CIT-TENG car dashboard vibration energy harvesting experiments were performed on the Hyundai Avante. The car was driven on a levelled off-road terrain with sand and pebble gravel (see Supporting Movie S3). The CIT-TENG water wave vibration energy harvesting experiments were performed using Jebao RW-8 Wavemaker pump. During this test the CIT-TENG was placed in an acrylic box wrapped with grounded aluminum foil in order to prevent electromagnetic noise from the pump from effecting the CIT-TENG output. The CIT-TENG machine vibration energy harvesting experiments were performed on the Airbank AB550 air compressor. The vibration acceleration levels from the car dashboard, water waves and air compressor were measured using an application on the Samsung Galaxy S8 mobile phone which employs an STMicroelectronics LSM6DSL inertial module. Fast fourier transform (FFT) of the measured vibration acceleration was taken using MATLAB to obtain the input frequency spectrum.

## Supplementary information

SUPPORTING INFORMATION

Supporting Movie S1

Supporting Movie S2

Supporting Movie S3

Supporting Movie S4
