# Supplementary material for: Continuous scavenging of broadband vibrations via omnipotent tandem triboelectric nanogenerators with cascade impact structure
Source: Sci Rep. 2019 Jun 3;9:8223. doi: 10.1038/s41598-019-44683-5 (PMC6547642; doi:10.1038/s41598-019-44683-5)
Supplement: Supplementary file 1 — SUPPORTING INFORMATION [file 41598_2019_44683_MOESM1_ESM.docx]

**SUPPORTING INFORMATION**

for

**Continuous scavenging of broadband vibrations via omnipotent tandem triboelectric nanogenerators with cascade impact structure**

Divij Bhatia^1^, Heejae Hwang^1^, Nghia Dinh Huynh^1^, Sangmin Lee^2^, Choongyeop Lee^1^, Youngsuk Nam^1^, Jin-Gyun Kim^1^, Dukhyun Choi^1^*

^1^Department of Mechanical Engineering, Kyung Hee University, 1732 Deogyeong-daero, Giheung-gu, Yongin-si, Gyeonggi-do 17104, South Korea.

^2^School of Mechanical Engineering, Chung-Ang University, 84 Heukseuk-ro, Dongjak-gu, Seoul 06974, South Korea.

*Email: dchoi@khu.ac.kr (Dukhyun Choi)

**
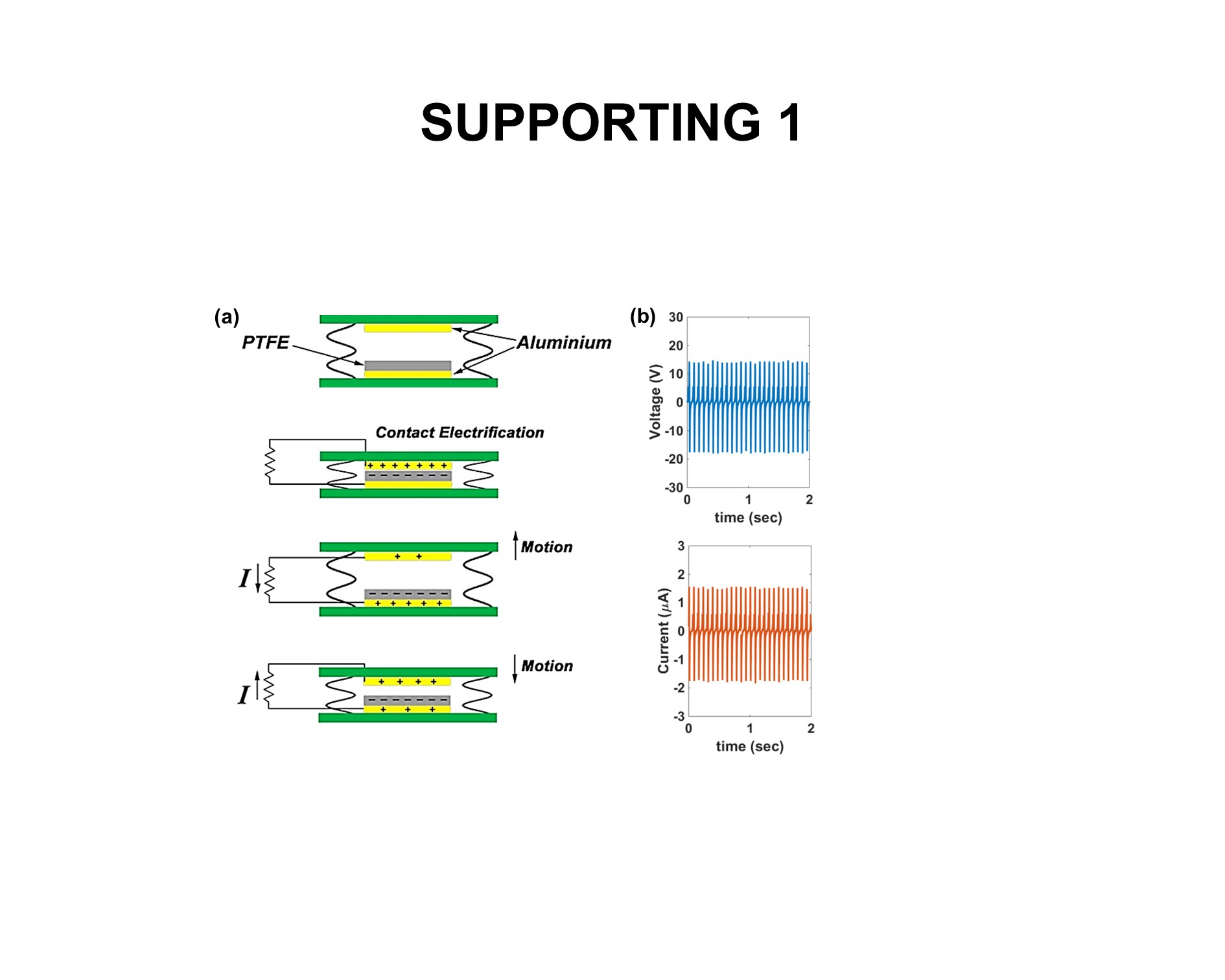
**

**Figure S1.** (a) Schematic showing cross-section and operation of a vertical contact separation mode vibration triboelectric nanogenerator. The bottom substrate was fixed to the vibration input source, while the top substrate was freely movable. PTFE and aluminum were repeatedly made to contact each other for several cycles in order to induce triboelectrification such that the PTFE developed stationary negative surface charges. When the aluminum moved away from the PTFE, conventional current flowed between the aluminum electrodes due to electrostatic induction. When the aluminum moved towards the PTFE, current flowed in the opposite direction. (b) Pulsed type alternating output from the TENG with respect to time, corresponding to the motion of the aluminum.

**Supporting Note 1:**

**Typical tandem TENG fabrication and assembly:** The typical tandem TENG was made up of four explicitly designed TENGs with target natural frequencies of 7 Hz, 20 Hz, 27 Hz, and 33 Hz. Accordingly, in order to achieve the target natural resonance frequencies for each of the TENGs, their mass and stiffness values were determined as 50 grams and 25*4 N/m for TENG-1, 11 grams and 40*4 N/m for TENG-2, 11 grams and 73*4 N/m for TENG-3, and 15 grams and 160*4 N/m for TENG-4, respectively. For each TENG, a polytetrafluoroethylene (PTFE) film of thickness 130 mm was used as the insulating dielectric material, and an aluminum film of thickness 16 μm was used as the top and bottom electrode material. The top electrode aluminum also served as the counter tribological contact material for static charging the PTFE surface. Effective contact area of the tribologically active top aluminum electrode and PTFE was 3.5 cm x 3.5 cm, similar to the CIT-TENG. The substrates of each TENG as well as the frame structure were 3D printed out of polylactic acid (PLA). Commercial double-sided tape (polyethylene (PE) foam) was used to fix the top and bottom aluminum electrodes to the PLA substrates. Four springs were used on each corner of the PLA substrates to support the TENG mass and ensure that a gap distance of 1 mm was maintained between the tribologically active materials in the initial state. The four TENGs were arranged in a stacked structure with length, width and height of the structure being 11 cm, 6.5 cm and 12 cm, respectively, as shown in Supporting Figure S2.

**
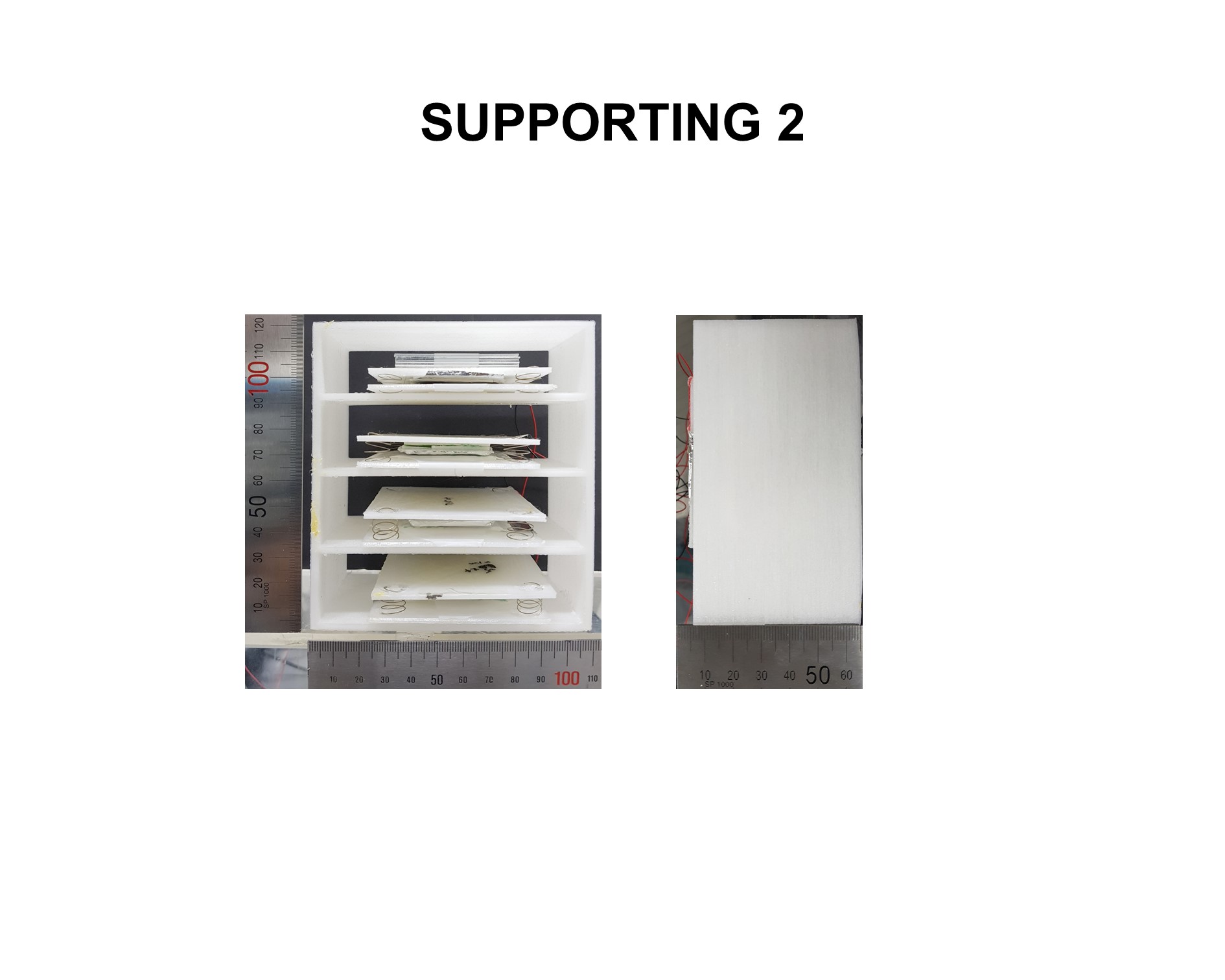
**

**Figure S2.** Photograph of typical stacked type tandem TENG used in this study and its dimensional measurements of 11 cm length, 12 cm height, and 6.5 cm width.

**
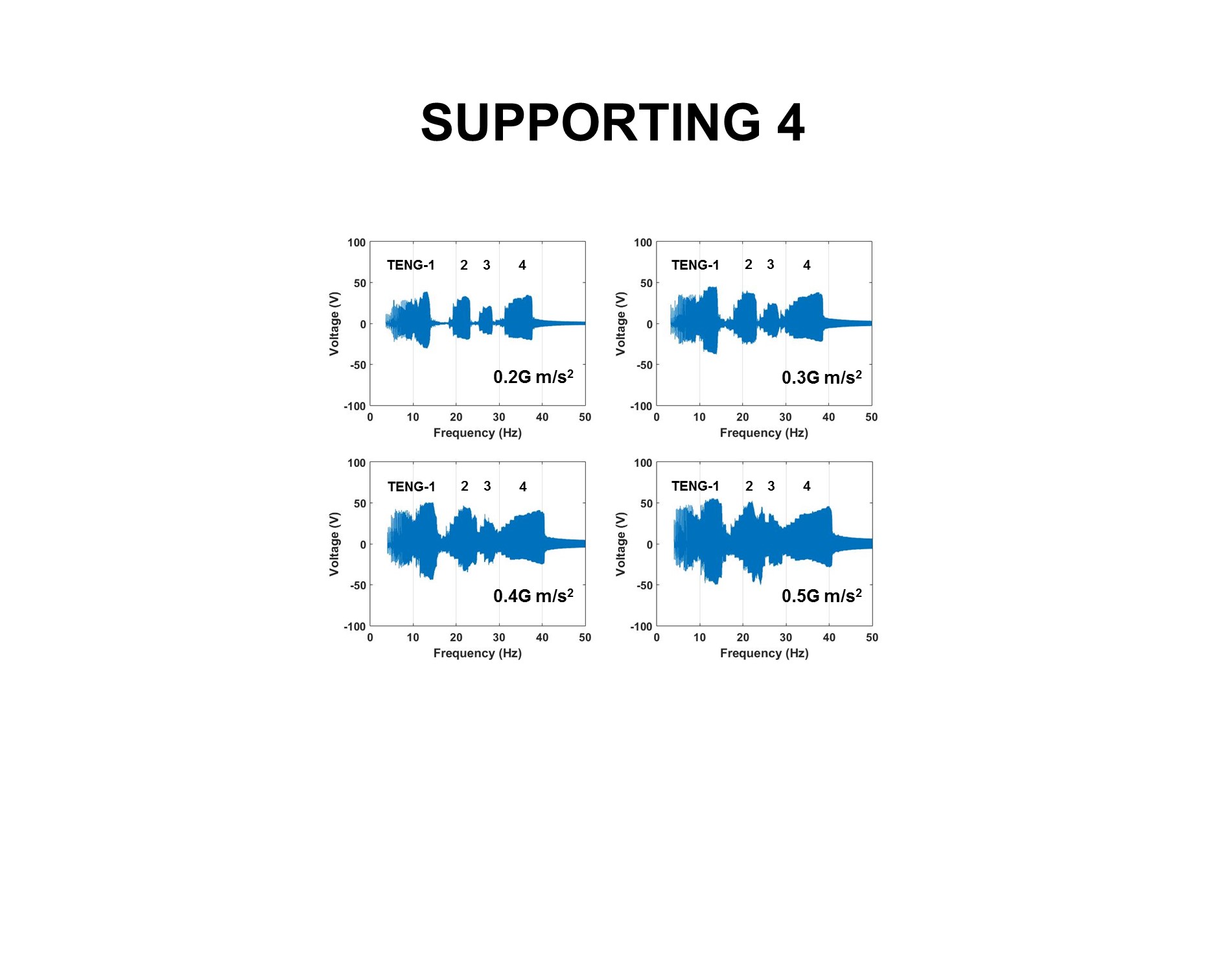
**

**Figure S3.** Experimental output voltage frequency response of the typical tandem TENG under low input accelerations of 0.2G m/s^2^ to 0.5G m/s^2^.

**
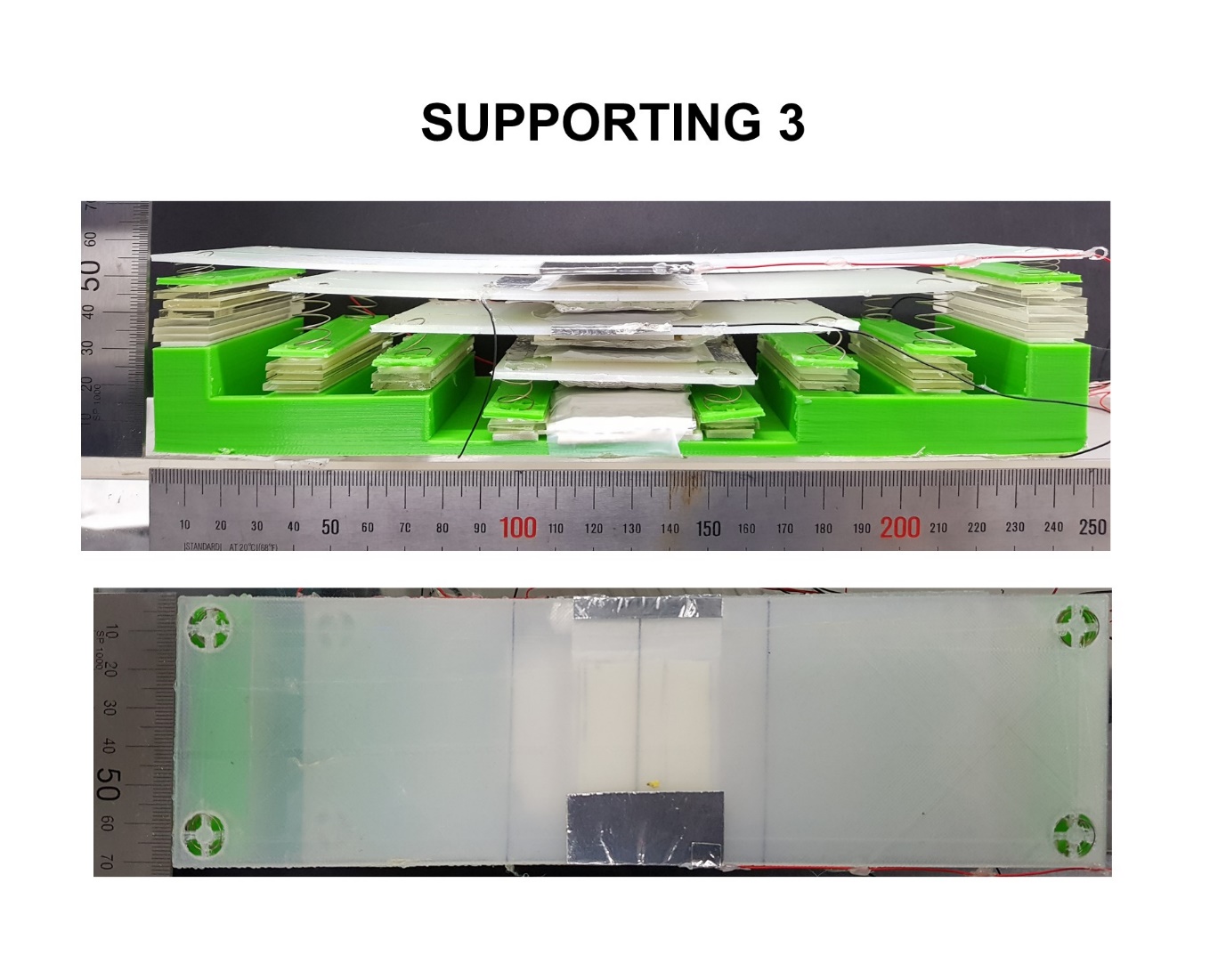
**

**Figure S4.** Photograph of CIT-TENG used in this study and its dimensional measurements of 25 cm length, 5.5 cm height, and 7 cm width.

**
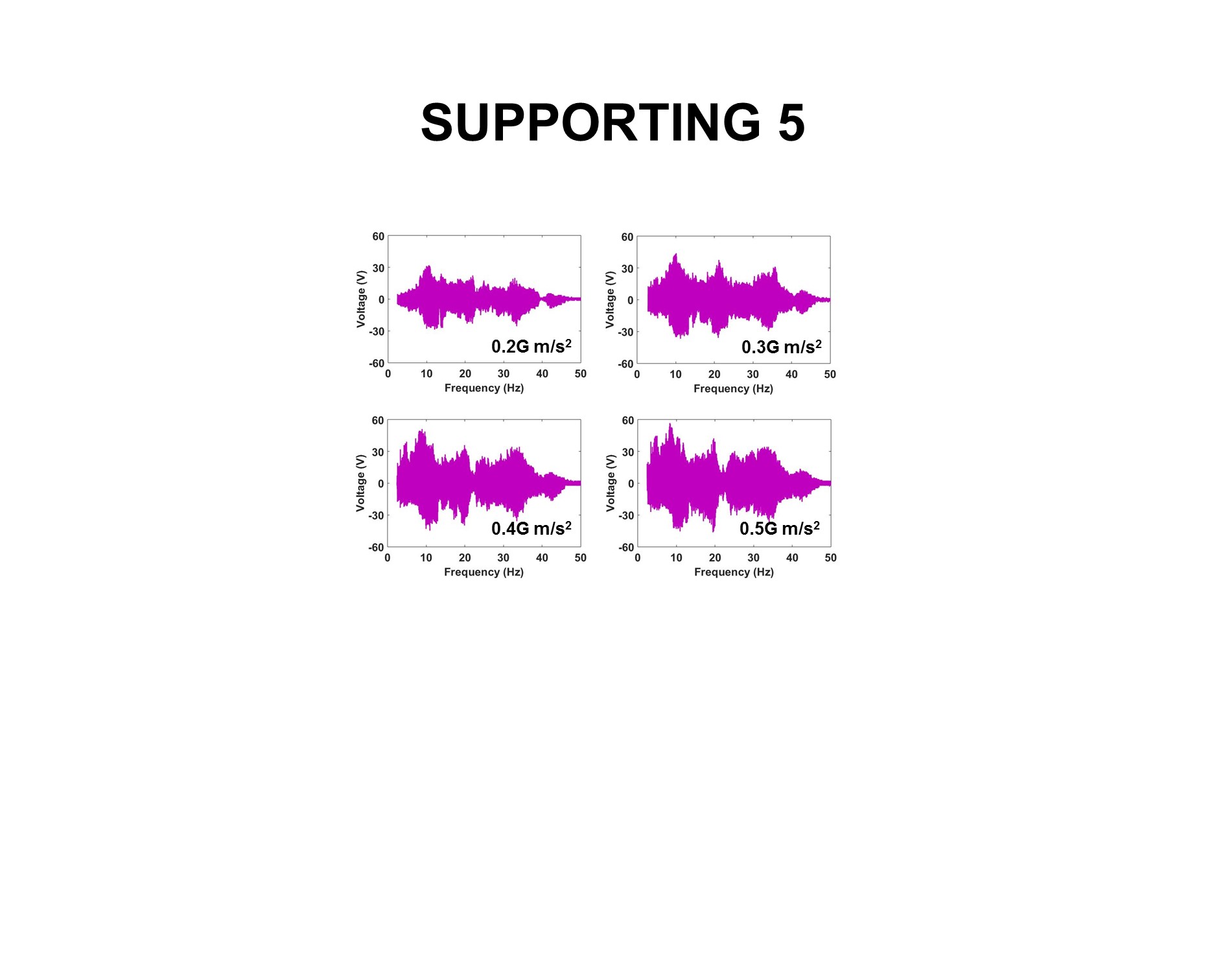
**

**Figure S5.** Experimental output voltage frequency response of the CIT-TENG under low input accelerations of 0.2G m/s^2^ to 0.5G m/s^2^.

**Supporting Note 2:**

**Vibration equations of motion for CIT-TENG:**

Considering TENG-1 impacting with TENG-2, if *m_1_, c_1_, k_1_* are the mass, damping, and stiffness of TENG-1, *m_2_, c_2_, k_2_* are the mass, damping, and stiffness of TENG-2, *d_12_* is the gap distance between TENG-1 and TENG-2, *y* is the input displacement, and *z_1_* is the displacement of mass *m*_1_, then the vibration equations describing non-impact state of TENG-1 and its impact state with TENG-2 can be given as,^13^

| $m_{1}\ddot{z_{1}}+c_{1}\dot{z_{1}}+k_{1}z_{1}=-m_{1}\ddot{y}$ | $-d_{12}<z_{1}$ | *No impact* |
| --- | --- | --- |
| $\left( m_{1}+m_{2} \right)\ddot{z_{1}}+\left( c_{1}+c_{2} \right)\dot{z_{1}}+\left( k_{1}+k_{2} \right)z_{1}-k_{2}d_{12}=-\left( m_{1}+m_{2} \right)\ddot{y}$ | $z_{1}\leq-d_{12}$ | *Impact with TENG-2* |

Similarly, if *m_3_, c_3_, k_3_* are the mass, damping, and stiffness of TENG-3, *d_23_* is the gap distance between TENG-2 and TENG-3, and *z_2_* is the displacement of mass *m*_2_, then TENG-2 vibration equations can be given as,

| $m_{2}\ddot{z_{2}}+c_{2}\dot{z_{2}}+k_{2}z_{2}=-m_{2}\ddot{y}$ | $-d_{23}<z_{2}<d_{12}$ | *No impact* |
| --- | --- | --- |
| $\left( m_{1}+m_{2} \right)\ddot{z_{2}}+\left( c_{1}+c_{2} \right)\dot{z_{2}}+\left( k_{1}+k_{2} \right)z_{2}-k_{1}d_{12}=-\left( m_{1}+m_{2} \right)\ddot{y}$ | $z_{2}\geq d_{12}$ | *Impact with TENG-1* |
| $\left( m_{3}+m_{2} \right)\ddot{z_{2}}+\left( c_{3}+c_{2} \right)\dot{z_{2}}+\left( k_{3}+k_{2} \right)z_{2}-k_{3}d_{23}=-\left( m_{3}+m_{2} \right)\ddot{y}$ | $z_{2}\leq{-d}_{23}$ | *Impact with TENG-3* |

Similarly, if *m_4_, c_4_, k_4_* are the mass, damping, and stiffness of TENG-4, *d_34_* is the gap distance between TENG-3 and TENG-4, and *z_3_* is the displacement of mass *m*_3_, then TENG-3 vibration equations can be given as,

| $m_{3}\ddot{z_{3}}+c_{3}\dot{z_{3}}+k_{3}z_{3}=-m_{3}\ddot{y}$ | $-d_{34}<z_{3}<d_{23}$ | *No impact* |
| --- | --- | --- |
| $\left( m_{2}+m_{3} \right)\ddot{z_{3}}+\left( c_{2}+c_{3} \right)\dot{z_{3}}+\left( k_{2}+k_{3} \right)z_{3}-k_{2}d_{23}=-\left( m_{2}+m_{3} \right)\ddot{y}$ | $z_{3}\geq d_{23}$ | *Impact with TENG-2* |
| $\left( m_{4}+m_{3} \right)\ddot{z_{3}}+\left( c_{4}+c_{3} \right)\dot{z_{3}}+\left( k_{4}+k_{3} \right)z_{3}-k_{4}d_{34}=-\left( m_{4}+m_{3} \right)\ddot{y}$ | $z_{3}\leq-d_{34}$ | *Impact with TENG-4* |

Similarly, if *c_s_, k_s_* are the damping, and stiffness of substrate material, *d_4s_* is the gap distance between TENG-4 and substrate, and *z_4_* is the displacement of mass *m*_4_, then TENG-4 vibration equations can be given as,

| $m_{4}\ddot{z_{4}}+c_{4}\dot{z_{4}}+k_{4}z_{4}=-m_{4}\ddot{y}$ | $-d_{4s}<z_{4}<d_{34}$ | *No impact* |
| --- | --- | --- |
| $\left( m_{3}+m_{4} \right)\ddot{z_{4}}+\left( c_{3}+c_{4} \right)\dot{z_{4}}+\left( k_{3}+k_{4} \right)z_{4}-k_{3}d_{34}=-\left( m_{3}+m_{4} \right)\ddot{y}$ | $z_{4}\geq d_{34}$ | *Impact with TENG-3* |
| $m_{4}\ddot{z_{4}}+\left( c_{s}+c_{4} \right)\dot{z_{4}}+\left( k_{s}+k_{4} \right)z_{4}-k_{s}d_{4s}=-m_{4}\ddot{y}$ | $z_{4}\leq{-d}_{4s}$ | *Impact with Substrate* |

**
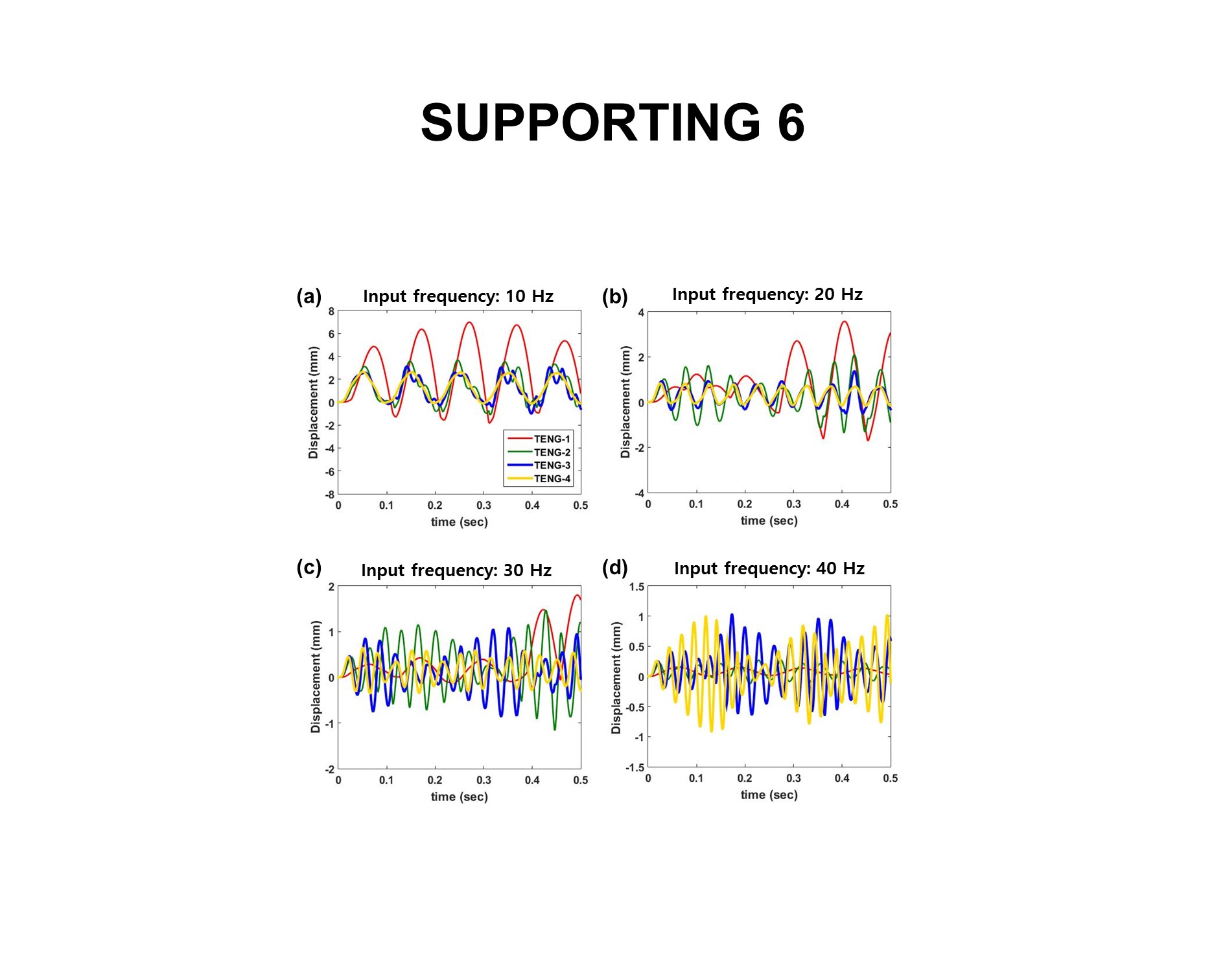
**

**Figure S6.** Simulated displacement time response of the TENGs at (a) 10 Hz, (b) 20 Hz, (c) 30 Hz, and (d) 40 Hz input frequencies.


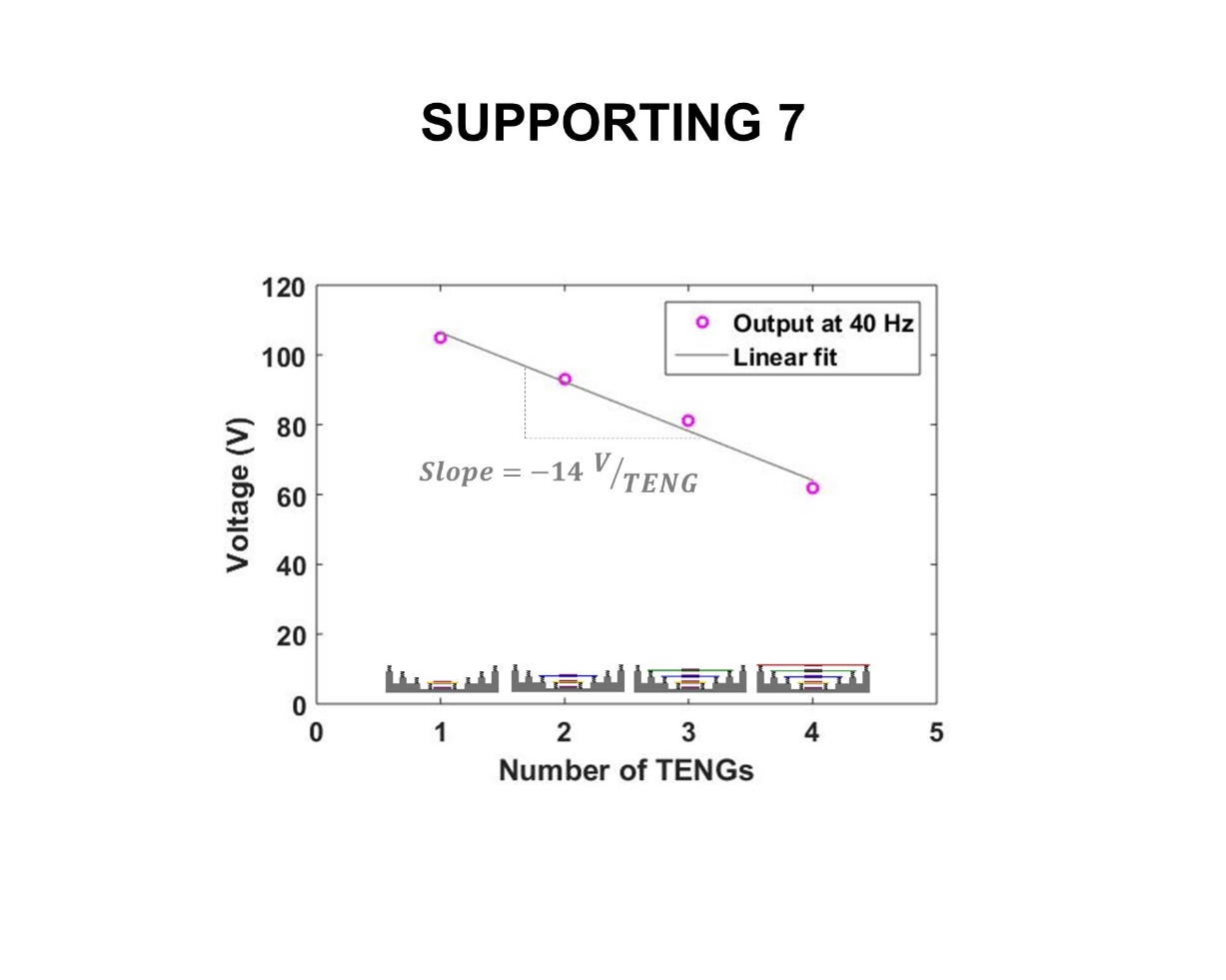


**Figure S7.** TENG-4 output voltage at 40 Hz input frequency as TENGs are installed layer by layer above it. Rate of voltage decrease from TENG-4 was 14V/TENG installed.


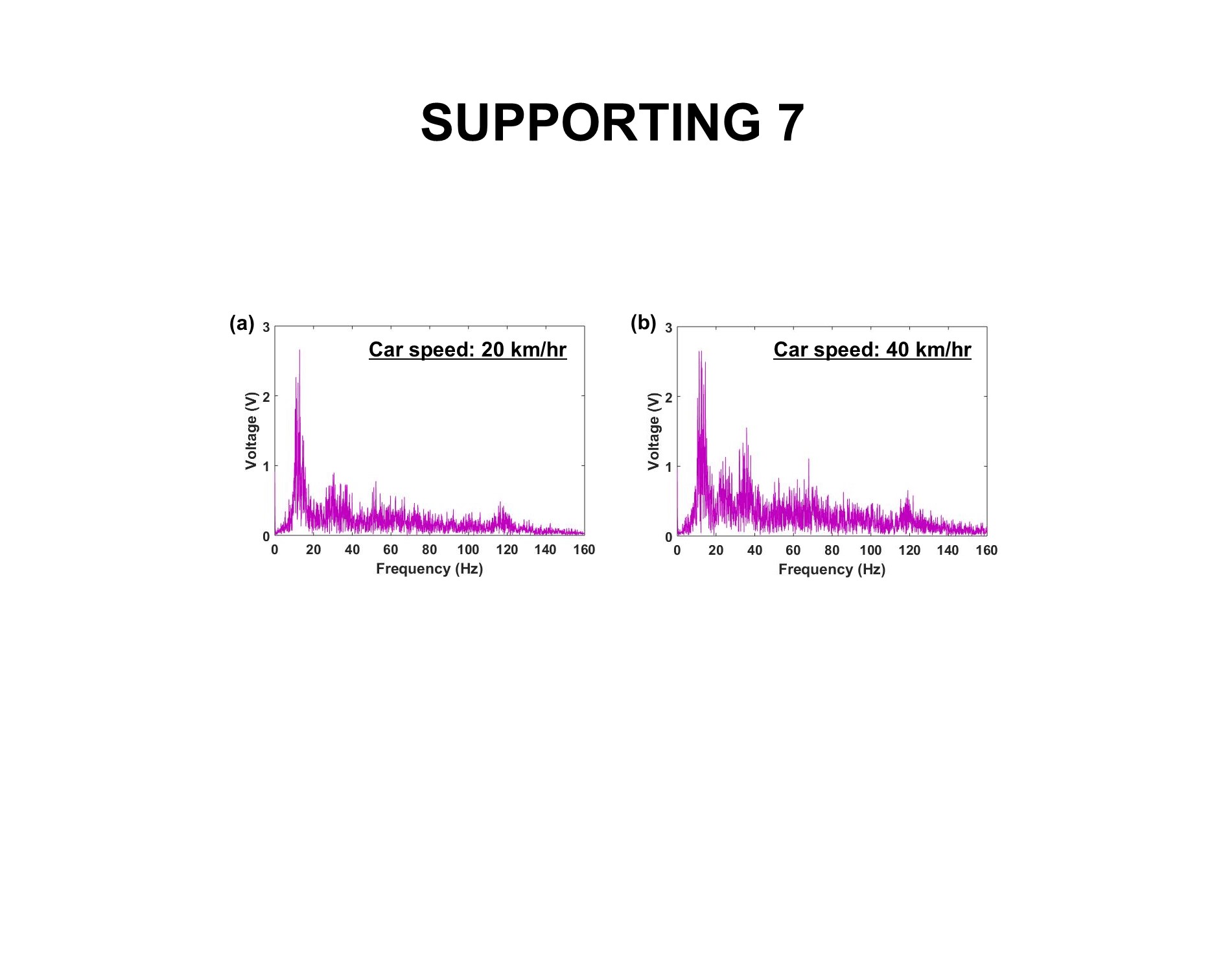


**Figure S8.** CIT-TENG output voltage frequency spectrums obtained by taking FFT of the voltage output at car speed of (a) 20 km/hr and (b) 40 km/hr.


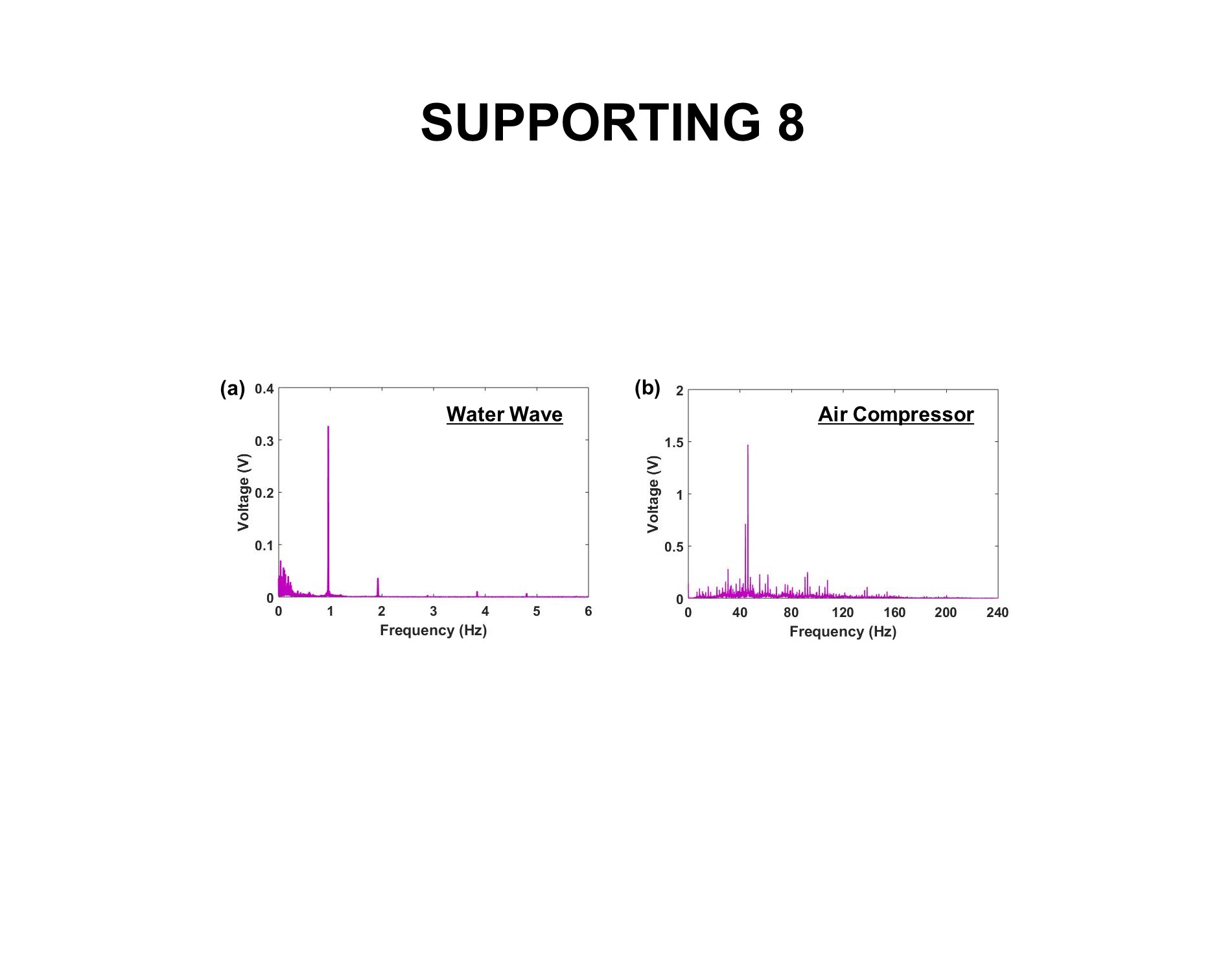


**Figure S9.** CIT-TENG output voltage frequency spectrums obtained by taking FFT of the voltage output from (a) water wave and (b) air compressor energy harvesting experiments.


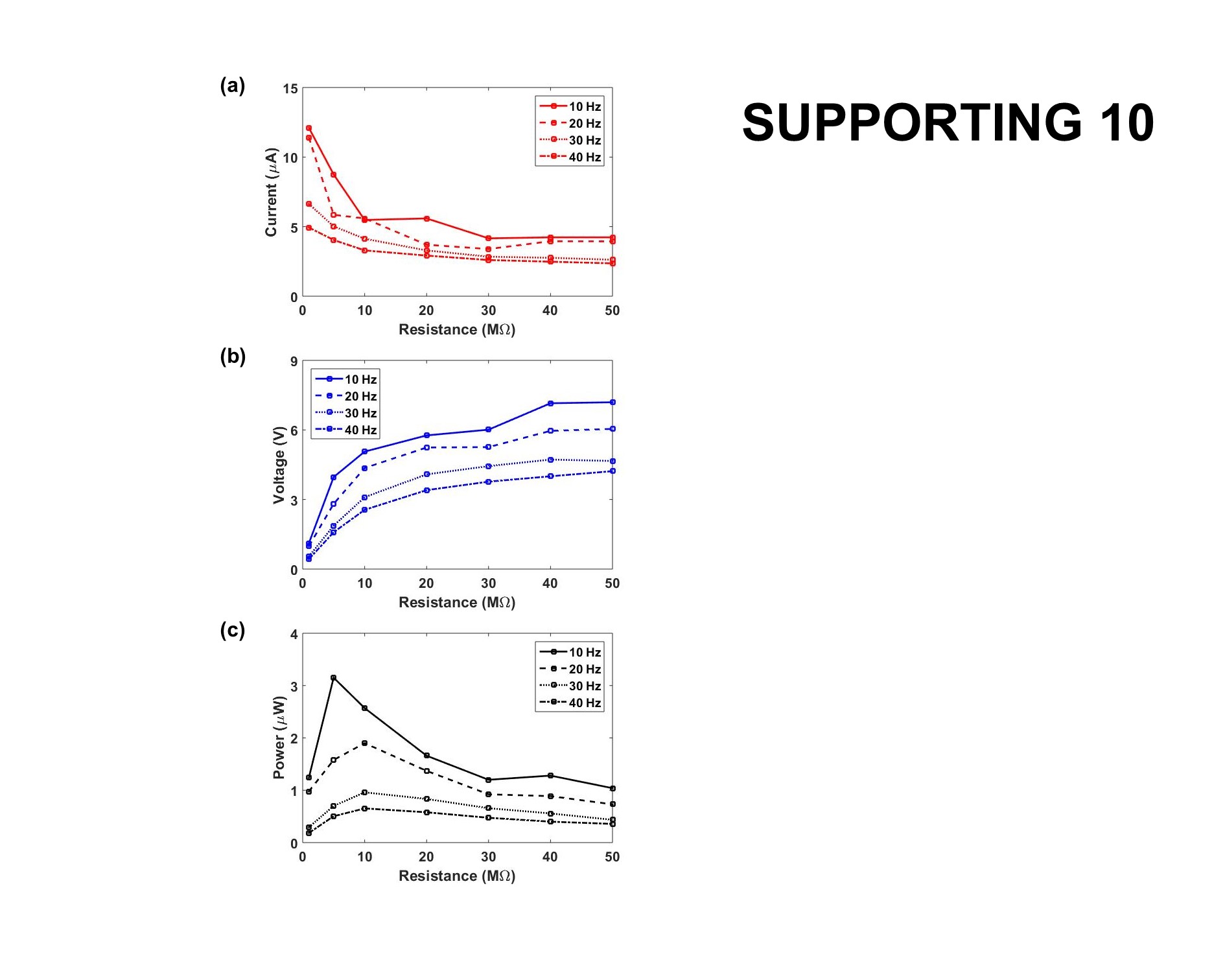


**Figure S10.** (a) Output current, (b) output voltage, and (c) output power from CIT-TENG under electrical loading conditions at 10 Hz, 20 Hz, 30 Hz, and 40 Hz input frequencies.

**Supporting Movie S1**

Video showing dynamic simulation of the CIT-TENG structure at sinusoidal input frequencies of 10 Hz, 20 Hz, 30 Hz, and 40 Hz.


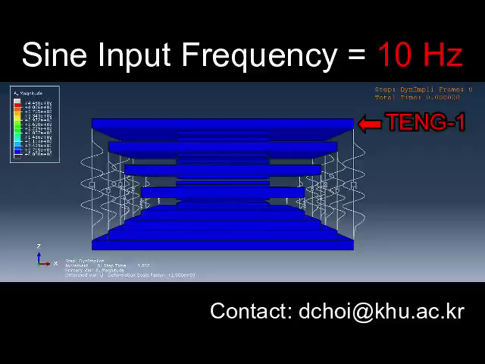


**Supporting Movie S2**

Video showing actual CIT-TENG vibration dynamics during frequency sweep testing.


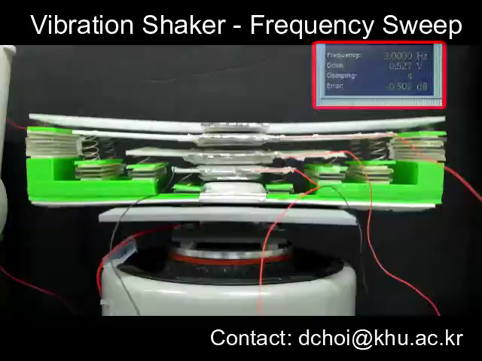


**Supporting Movie S3**

Video showing CIT-TENG vibration dynamics during car dashboard, water wave and air compressor application testing.


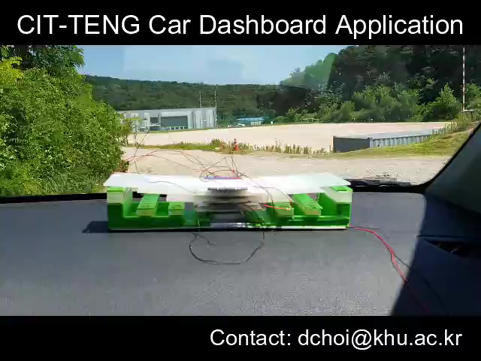


**Supporting Movie S4**

Video showing CIT-TENG directly powering LEDs when driven by air compressor. The LEDs can indicate that air compressor is in ON state.

~~
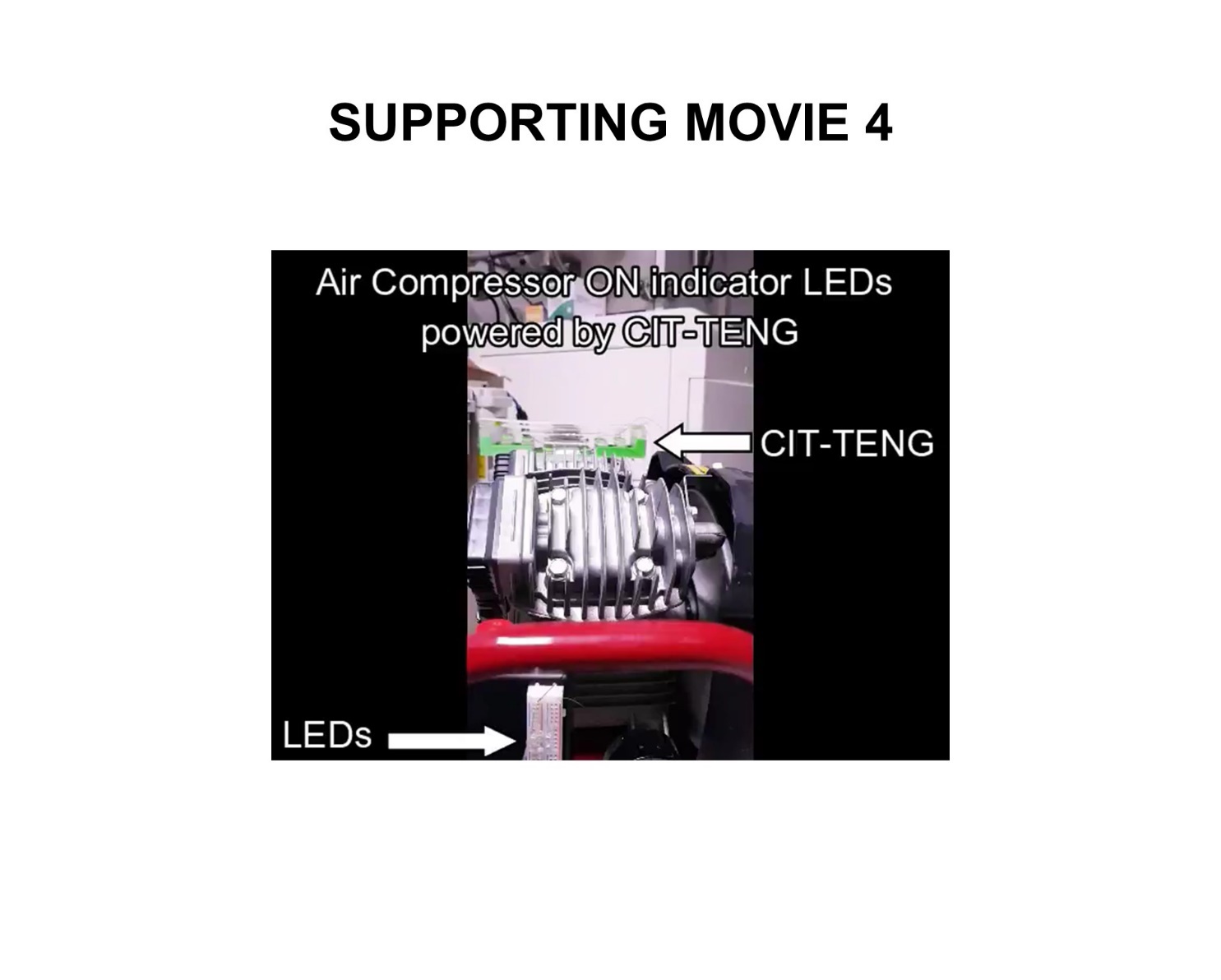
~~
